# Human stem cell-derived astrocytes exhibit region-specific heterogeneity in their secretory profiles

**DOI:** 10.1093/brain/awaa258

**Published:** 2020-09-08

**Authors:** Benjamin E Clarke, Doaa M Taha, Oliver J Ziff, Aftab Alam, Eric P Thelin, Núria Marcó García, Adel Helmy, Rickie Patani

**Affiliations:** a1 Department of Neuromuscular Diseases, Queen Square Institute of Neurology, University College London, London, UK; a2 The Francis Crick Institute, London NW1 1AT, UK; a3 Department of Zoology, Faculty of Science, Alexandria University, Alexandria 21511, Egypt; a4 Division of Neurosurgery and Wolfson Brain Imaging Centre, Department of Clinical Neurosciences, University of Cambridge, Cambridge, UK; a5 Department of Clinical Neuroscience, Karolinska Institute, Stockholm, Sweden

We recently reported in *Brain* that human induced pluripotent stem cell (iPSC)-derived astrocytes exhibit a distinct response to TDP-43 proteinopathy (both seeded aggregation and recombinant oligomers) compared to motor neurons. Furthermore, untreated control astrocytes were neuroprotective to motor neurons seeded with serially passaged post-mortem spinal cord tissue extracts derived from sporadic amyotrophic lateral sclerosis (ALS) cases (Smethurst *et al.*, 2020). Astrocyte-mediated neuroprotection was demonstrated in both physical co-cultures and by using astrocyte conditioned media, suggesting that factors released from astrocytes confer neuroprotection to motor neurons under pathological conditions. Human iPSC-derived astrocytes used in our published study had a ventral spinal cord identity, produced through the sequential application of an ontogeny-recapitulating programme of extrinsic cues including Wnt, retinoid and sonic hedgehog agonists as previously described (Hall *et al.*, 2017). These highly enriched cultures express astrocyte specific markers and display functional attributes including calcium sensitivity to ATP and increased production of several secreted factors in response to inflammatory stimulation (Hall *et al.*, 2017; Thelin *et al.*, 2020). Against this background, it is tempting to speculate that the cell type-specific phenotypes we described in our aforementioned *Brain* paper (Smethurst *et al.*, 2020), are applicable to the entire neuraxis. However, increasing recognition of region-specific functional heterogeneity among astrocytes (Clarke *et al*., 2020) raises the issue of whether these findings can safely be extrapolated to other regions of the nervous system such as the forebrain, which is also affected in ALS (upper motor neurons in layer V of the primary motor cortex and indeed more broadly in the recognized ALS-frontotemporal lobar degeneration spectrum disorders).

Indeed, regional heterogeneity of astrocytes is increasingly recognized as important to astrocytic function, providing unique support to juxtaposed neuronal subtypes (Kelley *et al.*, 2018; de Majo *et al.*, 2020). Since astrocytes play important roles in the pathomechanisms of several neurodegenerative diseases, understanding the potential differences in astrocyte populations from different regions may shed light on why particular neuronal populations are more susceptible to disease. Human iPSCs are a useful model to explore astrocyte heterogeneity as it has previously been shown that they can be positionally specified to different regions of the CNS using developmentally rationalized extrinsic cues following initial neural induction (Krencik *et al.*, 2011). More recently, functional differences in human iPSC-derived astrocytes patterned to different regions of the neuraxis have been described, with differences in their ability to stimulate the growth of co-cultured neurons and promote blood–brain barrier formation (Bradley *et al.*, 2019).

**Figure 1:**
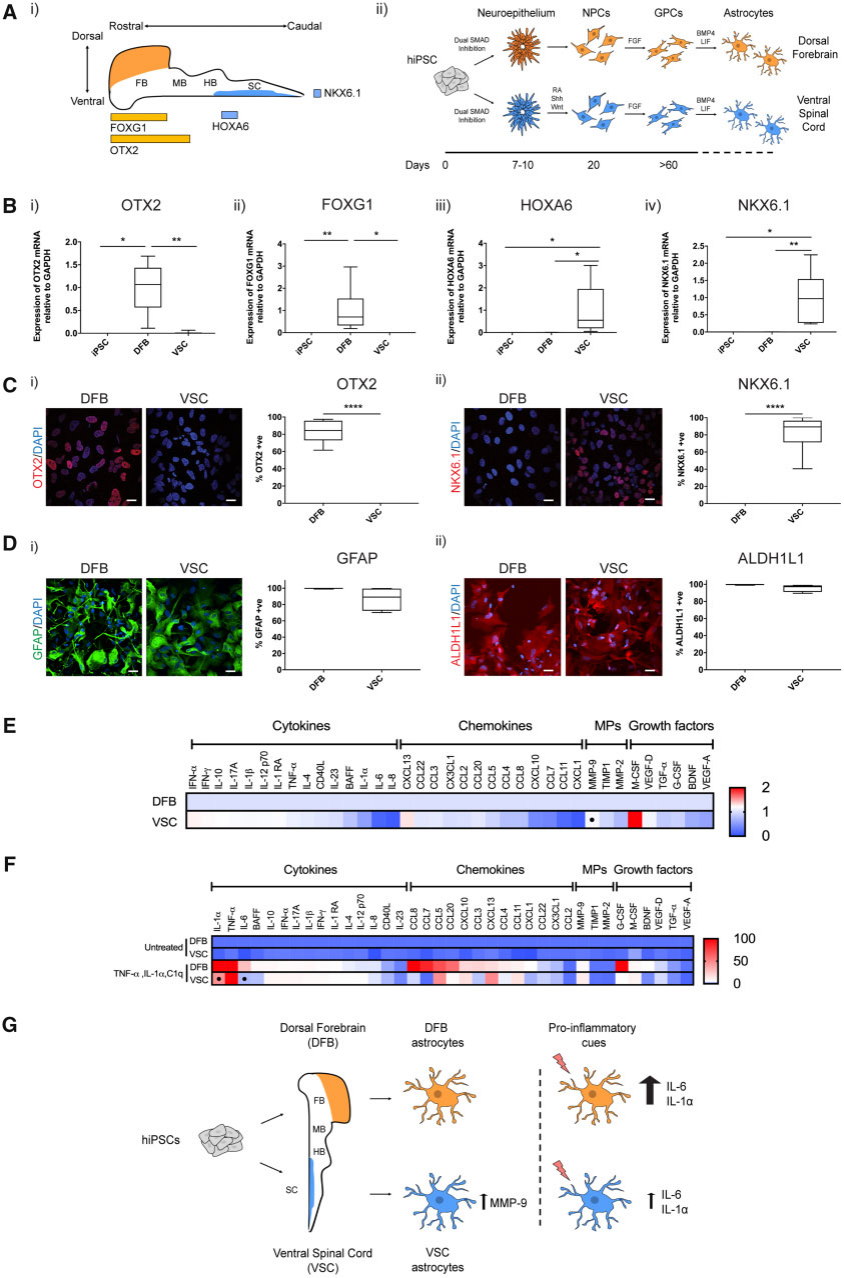
**Regionally distinct hiPSC-derived astrocytes exhibit differences in secreted factors.** [**A**(**i**)] Schema displaying regional markers in the developing nervous system. FB = forebrain; HB = hindbrain; MB = midbrain; SC = spinal cord. [**A**(**ii**)] Schema displaying protocol for directing the differentiation of human iPSCs to regionally different populations of astrocytes. GPCs = glial precursor cells; NPCs = neural precursor cells. (**B**) Quantitative PCR quantification of human iPSCs and NPCs patterned to the dorsal forebrain (DFB) or ventral spinal cord (VSC) for (**i**) FOXG1, (**ii**) OTX2, (**iii**) HOXA6 and (**iv**) NKX6.1. *n *=* *4 lines, one to two technical repeats. One-way ANOVA or Kruskal-Wallis test *P <* *0.05, **P <* *0.01. (**C**) Immunofluorescence and quantification of regional markers of NPCs for (**i**) OTX2 and (**ii**) NKX6.1. *n *=* *2 lines, seven fields per line imaged using a 510 Laser Scanning Confocal Microscope (Zeiss) and analysed using Fiji. Mann-Whitney U-test ****P <* *0.0001. Scale bar = 20 μm. (**D**) Immunofluorescence and quantification for astrocyte markers (**i**) GFAP and (**ii**) ALDH1L1. *n = *2 lines, two wells per line and 10 fields per well imaged using the Opera Phenix high content screening system (Perkin Elmer) and analysed using Columbus software (Perkin Elmer). Scale bar = 20 μm. (**E**) Luminex multiplex immunoassay (ThermoFisher) for DFB and VSC astrocytes under basal conditions displayed as fold change from DFB astrocytes. *n = *2 lines, two technical repeats. *P <* *0.05, Welch’s two-sample *t*-test. MPs = metallopeptidases. (**F**) Luminex multiplex immunoassay for DFB and VSC astrocytes treated with 100 ng/ml TNF-α, 100 ng/ml IL-1α and 1000 ng/ml C1q for 120 h displayed as fold change from untreated DFB astrocytes. *n = *2 lines, two technical repeats. *P <* *0.05, Welch’s two-sample *t*-test. (**G**) Schema depicting the establishment of regionally different astrocyte populations from human iPSCs and subsequent changes in the release of secreted factors.

To begin to address this issue in our model, we first generated and then compared regionally different populations of astrocytes by positionally specifying human iPSC-derived neural precursors to both the dorsal forebrain and ventral spinal cord using developmentally inspired extrinsic cues [Fig. 1A(i)]. We adapted previously described protocols to establish dorsal forebrain identity of human iPSC-derived neural precursors, which were then propagated *in vitro*, allowing them to undergo the temporally regulated gliogenic switch, before being terminally differentiated into astrocytes [Fig. 1A(ii)] (Kim *et al.*, 2011; Shi *et al.*, 2012; Bradley *et al.*, 2019). Neural precursors using this protocol expressed rostral neural tube markers OTX2 and FOXG1 [Fig. 1B(i and ii)]. Meanwhile we confirmed ventral spinal cord identity of our previously published protocol through the expression of spinal marker HOXA6 and ventral marker NKX6.1 [Fig. 1B(iii and iv)] (Hall *et al.*, 2017). This was also confirmed through quantitative immunofluorescence, which showed that on average more than 80% of cells patterned to either the dorsal forebrain or ventral spinal cord expressed OTX2 or NKX6.1, respectively (Fig. 1C).

Both protocols produced highly enriched populations of human astrocytes upon terminal differentiation, identified by the expression of intermediate filament marker GFAP or pan astrocyte marker ALDH1L1 (Fig. 1D). To assess whether there were differences between factors that are released from astrocytes patterned to these two regions, we used a Luminex multiplex immunoassay (Thermo Fisher) to measure the release of several important cytokines, chemokines, metallopeptidases and growth factors in astrocyte conditioned media. Under basal conditions, several factors were differentially secreted from ventral spinal cord astrocytes compared with dorsal forebrain astrocytes (Fig. 1E), of which MMP9, a metallopeptidase that has been linked to the selective vulnerability of fast firing motor neurons in ALS (Kaplan *et al.*, 2014), was found to be significantly increased in ventral spinal cord astrocytes (*P *=* *0.049, Welch’s two-sample *t*-test). In addition, several other factors were either increased (M-CSF and CXCL13) or decreased (IL-6, IL-8 and BDNF) in ventral spinal cord astrocytes, although these did not reach statistical significance.

Astrocytes are able to undergo dramatic changes to their morphology, gene expression and function in response to different stimuli in a process termed astrocyte reactive transformation (Escartin *et al.*, 2019), which has been associated with the pathomechanisms of several neurodegenerative diseases (Liddelow and Barres, 2017). Therefore, we next stimulated dorsal forebrain and ventral spinal cord astrocytes with a combination of established pro-inflammatory factors (Liddelow *et al*., 2017) to investigate potential differences in secretory responses of astrocytes from these different regions. TNF-α, IL-1α and C1q treatment resulted in a large increase in many factors in both forebrain and ventral spinal cord derived astrocyte conditioned media (Fig. 1F). Several of these were upregulated to a greater degree in dorsal forebrain astrocytes compared to ventral spinal cord astrocytes, including IL-1α (*P *=* *0.002) and IL-6 (*P *<* *0.001, two-way ANOVA with Tukey’s *post hoc* tests), with BAFF, CCL3, CCL5, CCL7, CCL8, G-CSF and BDNF all following a similar trend, suggesting that regional differences exist in the responses of human iPSC-derived astrocytes to reactive stimuli.

In summary, these data show that human iPSC-derived astrocytes positionally specified to different regions of the neuraxis display differences in both their basal and stimulated secretomes including a diverse range of cytokines, chemokines, metallopeptidases and growth factors (Fig. 1G). These data suggest that astrocyte heterogeneity extends to the secretion of factors, which is likely to have important implications for surrounding neurons in these regions. It follows that regional differences in the reactivity of astrocytes may have implications for different neurodegenerative diseases, each characterized by region-specific neuronal loss . These results raise the possibility that astrocyte dysfunction in the ventral spinal cord affecting lower motor neurons may differ from astrocyte dysfunction in the cerebral cortex affecting upper motor neurons, although further studies will be needed to confirm this hypothesis.

##  

### Data availability

The data that support the findings of this study are available from the corresponding author, upon reasonable request.
